# Influence of Emulsified Asphalt on the Mechanical Property and Microstructure of Cement-Stabilized Gravel under Freezing and Thawing Cycle Conditions

**DOI:** 10.3390/ma10050504

**Published:** 2017-05-06

**Authors:** Yiqi Wang, Yiqiu Tan, Meng Guo, Xinglong Wang

**Affiliations:** 1 School of Transportation Science and Engineering, Harbin Institute of Technology, Harbin 150090, China; wangyiqihit@163.com; 2 College of Civil and Architectural Engineering, Heilongjiang Institute of Technology, Harbin 150050, China; 3 National Center for Materials Service Safety, University of Science and Technology Beijing, Beijing 100083, China; 4 Heilongjiang Institute of Highways and Transport Research, Harbin 150080, China; xinglongwang@163.net

**Keywords:** emulsified asphalt, cement-stabilized gravel, freezing and thawing cycle, mechanical property, microstructure, frost-resistance property

## Abstract

Properties of cement-stabilized gravel modified by emulsified asphalt under freezing and thawing cycle conditions were investigated by adjusting the dosage of cement. Mercury intrusion porosimetry (MIP) and Scanning electron microscopy (SEM) were introduced to analyze the influential mechanism. The results indicate that cement emulsified asphalt stabilized gravel with 5 wt % of cement performed well in both mechanics and frost-resistance. Although the addition of emulsified asphalt would lead to a partial decrease of strength, it can extend the process of strength loss and improve the freezing resistance. The main reason for this is that the permeability can be improved by the filling effects of emulsified asphalt. The frost-heave stress caused by the phase transition of water can also be remitted by emulsified asphalt, the elasticity modulus of which is much lower than the matrix. The generating speed of the micro crack can also be slowed down by emulsified asphalt.

## 1. Introduction

To adapt to requirements for national economic development, the structural form of semi-rigid base has been successfully proposed through a large quantity of research and extensive application in China. Semi-rigid base materials have advantages, e.g., favorable plank body performance, high bearing capacity, convenient utilization of local materials, low cost, etc., which have provided a reliable guarantee for realizing “strong base and thin surface” asphalt pavement structure. Benefiting from these advantages, semi-rigid base materials account for 95% of highway base materials in China. The cement-stabilized gravel (CSG) is being used widely as a typical semi-rigid base material [1,2,3,4]. On account of its aggregate gradation and hydration of the cementing material, CSG contains a considerable number of pores in its structure [5,6]. A certain number of cracks will develop in a CSG base once it is laid, forming open channels [7]. When a CSG base is used in a pavement structure in a cold or severely cold region, the water present in the open pore channels in the base structure will repeatedly undergo phase changes at low temperature and consequently expand under freeze–thaw cycling, resulting in damage inside the structure [8]. This in turn results in a decline in the strength and durability of the base. Consequently, the pavement will sustain damage to its entire structure under loading during its service life, resulting in safety issues and loss of invested capital [9].

Freeze-thaw cycles could significantly influence the performance of CSG by damaging the interfacial adhesion between different materials [10,11]. Yan et al. investigated the effects of freeze–thaw cycles on the characteristics of Stone Mastic Asphalt (SMA) mixtures containing different contents of cement. They found that using lime to replace cement could improve the freeze–thaw resistance of SMA mixtures [12]. Hazaree et al. studied the effects of variations in the cement content and air entrainment on basic physical, mechanical properties and freeze–thaw resistances of roller compacted concrete mixtures. They found that the air entrainment could affect the strength and freeze-thaw durability of the mixes [13]. Michael and Takafumi studied the effect of freezing-thawing action on internal structure of mortars by using the X-ray microtomography. They found that the initial air voids follow a lognormal distribution with the highest population of modal size around 30–50 μm. This was independent of the type of mortar [14].

One effective means to improve the performance of CSG is to modify the cementing material. The use of emulsified asphalt (EA) to improve the material’s flexibility is one area of research that has received considerable attention [15,16]. Miljković and Radenberg studied the effect of EA on the asphalt mixture performance and analyzed the fracture mechanism [17,18]. The results implied the potential of EA to improve the freeze-thaw resistance of CSG. Mixing CSG, which possesses plate material-like properties [19], with EA imparts CSG with some flexibility and inhibits it from shrinking, thereby reducing structural defects [20,21]. Regarding the CSG and EA mixture, researchers have mostly focused on the effect that EA has on the shrinking and mechanical performance and wear resistance of CSG [22,23,24,25,26,27]. The objective of this study was to investigate the effect of freeze-thaw on the mechanical property and microstructure of CSG, and to analyze the improvement of EA on the freeze-thaw resistance of CSG.

## 2. Materials and Methods

### 2.1. Raw Materials

**Figure 1:**
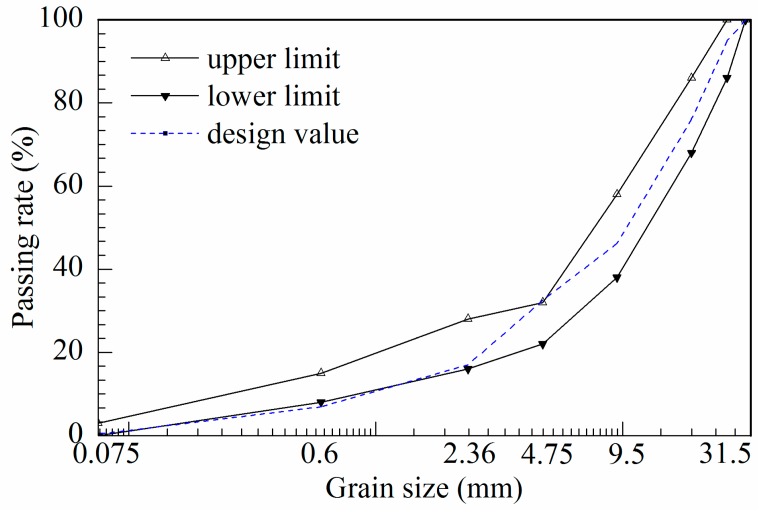
Grading curves of the cement-stabilized macadam.

Ordinary Portland cement P.O42.5 was chosen in this research, and its properties are shown in Table 1. The technical specifications of the aggregates used in the semi-rigid base material are shown in Table 2, and the grading curve is shown in Figure 1. The cationic slow-cracking EA was used in the experiment. Table 3 lists the basic properties of the selected EA.

### 2.2. Specimen Preparation and Curing

A previous study demonstrated that relatively good modification results were achieved with an EA content of 2–3%. Therefore, an EA content of 2.5% was used in the present study. Table 4 lists the cement and EA contents in the CSG specimens prepared in the present study.

According to the Testing Procedure for Inorganic Binder-stabilized Materials for Highway Construction (JTG E51-2009), cylindrical specimens (Φ150 mm × 150 mm) were prepared using the static pressure method based on a predetermined maximum dry density and optimal water content. For each mixing ratio, nine specimens were prepared for comparison. In the present study, the mechanical performance of the EA-modified CSG was examined under three different freeze–thaw cycles (i.e., 5, 10 and 15). For each freeze–thaw cycle, nine specimens were prepared. Specimens were removed from the molds 1 d (Does d represent days) after the molding process. Subsequently, measurements were taken to determine the height, diameter and weight of each specimen.

Afterward, the specimens were placed in plastic bags and cured in a standard curing chamber.

### 2.3. Experimental Method

#### 2.3.1. Freeze–Thaw Cycling Test

After 28 days of curing, the specimens were immersed and saturated in 20 ± 2 °C water for 24 h. The water level was higher than the specimens by 2.5 cm. Subsequently, the surfaces of the specimens were dried, and then, the specimens were frozen in a low-temperature (−18 °C) freezer for 16 h. The specimens were then thawed in a 20 °C water bath for 8 h. This completed one freeze–thaw cycle. After the specimens had undergone the preset number of freeze–thaw cycles, their surfaces were dried with a soft cloth and then subjected to mechanical testing.

#### 2.3.2. Unconfined Compressive Strength Testing

Each specimen that had undergone the preset number of freeze–thaw cycles was subjected to compressive loading at a rate of 1 mm/min using a universal testing machine. The maximum pressure, *F* (N), when the specimen failed was recorded. In addition, the representative value of the unconfined compressive strength of the specimen, *P* (MPa), was also calculated. The number of replicate samples is 13 at each amount of cement and emulsified asphalt.

#### 2.3.3. Dynamic Compressive Resilient Modulus Testing

According to the Laboratory Testing Method for Compressive Resilient Modulus of Inorganic Binder-stabilized Materials (T0857-2009) detailed in the Testing Procedure for Inorganic Binder-stabilized Materials for Highway Construction (JTG E51-2009), dynamic compressive resilient modulus testing was conducted using a UTM-250. The number of replicate samples is 9 at each amount of cement and emulsified asphalt.

#### 2.3.4. Pore Structure Testing and Microstructure Analysis

**Figure 2:**
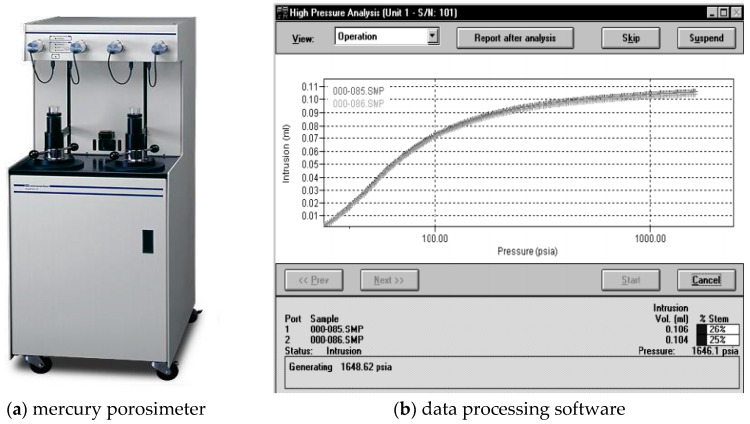
Mercury intrusion porosimetry (MIP) and software interface.

Each specimen for testing was crushed into particles with a size ranging from 8 to 10 mm, which were then vacuum-dried at 25 °C for 12 h. Afterward, sampling was performed on each specimen. Pore structure testing was conducted on an AUTOPOREIV 9500 mercury porosimeter (pore size measurement range: 360 μm–5.5 nm) manufactured by Micromeritics (Norcross, GA, USA). The instrument and data processing software are shown in Figure 2. Each collected specimen was fixed and sprayed with gold, and then, its micromorphology was analyzed by using a scanning electron microscope (FEI Quanta200, FEI, HL, USA).

## 3. Results and Discussion

### 3.1. Unconfined Compressive Strength Testing

**Figure 3:**
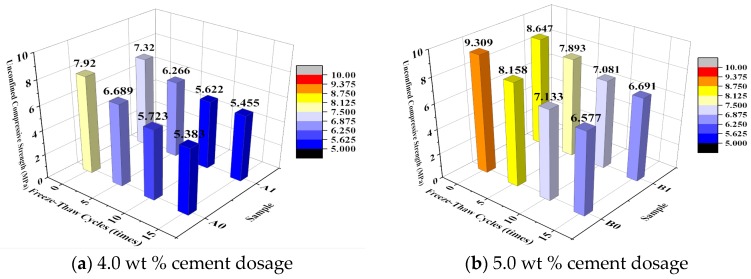
Influence of freeze-thaw cycles on unconfined compressive strength.

**Figure 4:**
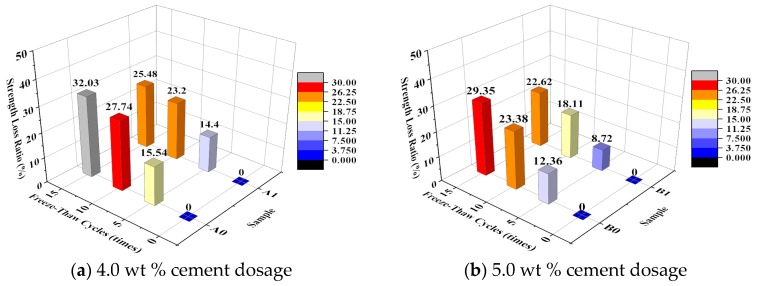
Relationship between freeze-thaw cycles and strength loss ratio.

Figure 3 shows the effect of the number of freeze–thaw cycles on the unconfined compressive strength of the specimen. Figure 4 shows the relationship between the number of freeze–thaw cycles and the strength loss ratio. The variation coefficients are calculated at each amount of cement and emulsified asphalt, and shown in Table 5.

Based on Figure 3, the unconfined compressive strength of the specimens of each group decreased with increasing number of freeze–thaw cycles, regardless of whether the specimens were modified with EA. The addition of EA resulted in a decrease in the initial strength of the CSG to a certain extent. In addition, the strength of the CSG with a cement content of 5.0% decreased to a relatively small extent. After 5 and 10 freeze–thaw cycles, the unconfined compressive strength of all the EA-modified specimens was lower than that of the unmodified specimens. However, after 15 freeze–thaw cycles, the unconfined compressive strength of the EA-modified specimens was higher than that of the unmodified specimens.

As seen in Figure 4, higher cement contents resulted in lower strength loss ratios following the freeze–thaw cycles. The strength loss ratio of all the specimens with a cement content of 4.0% was relatively high (>14%) after 5 freeze–thaw cycles but decreased as the number of freeze–thaw cycles increased. For each specimen with a cement content of 5.0%, the highest strength loss occurred after the specimen underwent 5–10 freeze–thaw cycles. It can be seen from Table 5 that the tests results are acceptable because of the small variation coefficients.

For the EA-modified specimens, the trend of strength loss became stable as the number of freeze–thaw cycles increased. In addition, after 15 freeze–thaw cycles, the strength of the EA-modified specimens was higher than that of the unmodified specimens. Therefore, while the addition of EA resulted in a decrease in the initial strength of the CSG to a certain extent, this modification improved the frost resistance of the CSG and slowed its strength loss process.

### 3.2. Dynamic Compressive Resilient Modulus Testing

**Figure 5:**
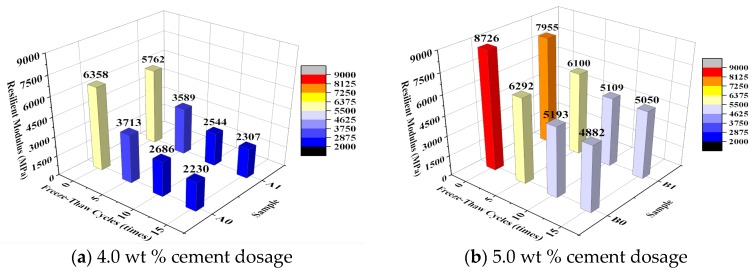
Influence of freeze-thaw cycles on dynamic compressive resilient modulus.

**Figure 6:**
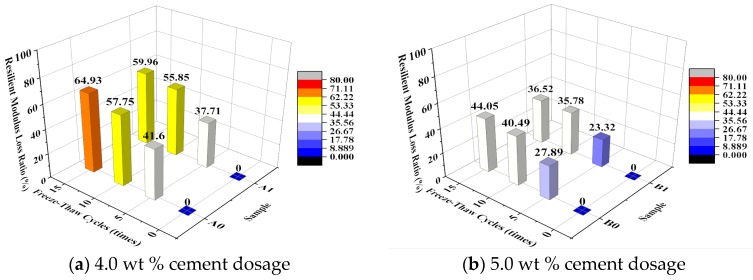
Relationship between freeze-thaw cycles and compressive resilient modulus loss ratio.

Figure 5 shows the effect of the number of freeze–thaw cycles on the dynamic compressive resilient modulus of the specimen. Figure 6 shows the relationship between the number of freeze–thaw cycles and the dynamic compressive resilient modulus loss ratio. The variation coefficients are calculated at each amount of cement and emulsified asphalt, and shown in Table 6.

Based on Figure 5, the dynamic compressive resilient modulus of the specimens of each group decreased as the number of freeze–thaw cycles increased. As a component that provides flexibility, EA, when mixed in CSG, resulted in an increase in the flexibility of the CSG system within a small range and a decrease in its dynamic compressive resilient modulus to a small extent. After 5 and 10 freeze–thaw cycles, the dynamic compressive resilient modulus of all the EA-modified specimens was lower than that of the unmodified specimens. However, after 15 freeze–thaw cycles, the dynamic compressive resilient modulus of all the EA-modified specimens was higher than that of the unmodified specimens. Of all the specimens, the EA-modified specimens with a cement content of 5.0% retained their dynamic compressive resilient modulus at the highest percentages after the freeze–thaw cycles.

As seen in Figure 6, the dynamic compressive resilient modulus loss ratio decreased significantly under the same number of freeze–thaw cycles as the cement content increased. The dynamic compressive resilient modulus loss ratio of the specimens with a cement content of 5.0% after 10 freeze–thaw cycles (approximately 40%) was close to that of the specimens with a cement content of 4.0% after 5 freeze–thaw cycles. The dynamic compressive resilient modulus loss ratio of the specimens with a cement content of 4.0% after 10 freeze–thaw cycles reached nearly 60%. A comparison of the EA-modified and unmodified specimens with the same cement content shows that the addition of EA resulted in a decrease in the dynamic compressive resilient modulus loss ratio. The small variation coefficients in Table 5 indicate that the tests results are acceptable.

By analyzing the effect of freeze–thaw cycling on the unconfined compressive strength and dynamic compressive resilient modulus, it is found that under freeze–thaw cycling, although the introduction of EA reduced the initial dynamic compressive resilient modulus of the CSG, this modification increased the percentage of its dynamic compressive resilient modulus as the number of freeze–thaw cycles increased and improved its frost resistance. The EA-modified CSG specimens with a cement content of 5.0% outperformed all the other specimens in terms of macroscopic mechanical performance and frost resistance.

### 3.3. Pore Structure Testing and Microstructure Analysis

#### 3.3.1. CSG Pore Structure and Microstructure

**Figure 7:**
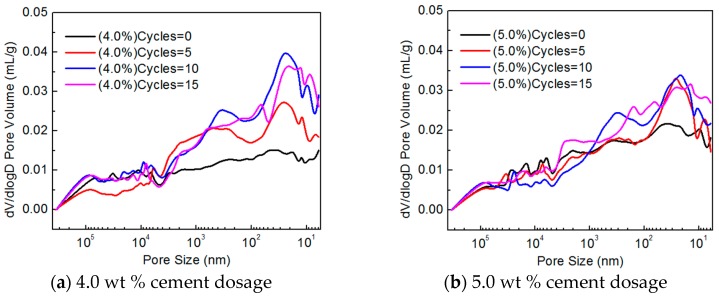
Pore size distribution of cement-stabilized gravel.

Figure 7 shows the pore size distributions of CSG specimens with various cement contents. As seen in Figure 7a for the CSG specimens with a cement content of 4.0%, the number of pores larger than 5 µm in size first decreased and then increased with increasing number of freeze–thaw cycles, which is the collective result of cement hydration and freeze–thaw-induced damage. After the freeze–thaw cycling started, water entered the existing pores in the structure, which stimulated some of the unhydrated cement particles to undergo hydration. Overall, this caused an increase in the compactness of the structure by reducing the number of large pores and increasing the number of small pores. Additionally, this step also caused damage to the structure as a result of the phase change (freezing)-induced expansion of the water, albeit to a relatively small extent. Pores larger than 5 µm in size were mainly marked by their high density and reduced numbers. As the number of freeze–thaw cycles increased, the number of unhydrated particles decreased. Damage resulting from the phase change (freezing)-induced expansion of the water played a dominant role, and the number of pores in the structure increased. Therefore, the number of pores larger than 5 µm in size decreased after 5 freeze–thaw cycles but increased after 10 freeze–thaw cycles. The number of pores smaller than 1 µm in size increased with increasing number of freeze–thaw cycles. This is because pores within this size range were mostly distributed around nearly complete hydration products and the number of pores increased with increasing freeze–thaw-induced damage.

For the CSG with a cement content of 5%, the number of pores larger than 5 µm in size decreased with increasing number of freeze–thaw cycles within 10 freeze–thaw cycles (Figure 7b), and the reaction mechanism was consistent with that for the CSG with a cement content of 4.0%; however, the proportion of unhydrated particles increased, and the duration of the dominant compacting effect of hydration on the structure also increased. The number of pores smaller than 1 µm in size increased as the number of freeze–thaw cycles increased because of damage induced by the freeze–thaw cycles.

**Figure 8:**
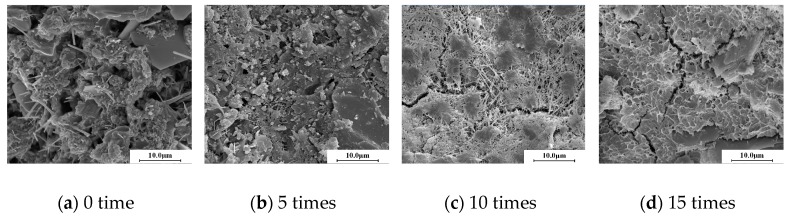
Microstructure of cement-stabilized gravel.

Figure 8 shows the microstructure of the CSG specimens with a cement content of 5.0%. Their micromorphology shows that as the number of freeze–thaw cycles increased, the structure became more compact and a relatively large number of cracks formed because of freezing-induced expansion; in addition, the cracks at the aggregate–cementing material interface were more visible, and the CSG structure deteriorated and sustained damage.

Based on the pore structure test results and microstructure analysis, the CSG specimens modified with various EA contents exhibited the following trend: The porosity increased and the compactness of the structure decreased as the number of freeze–thaw cycles increased. However, the specimens with a relatively high cement content sustained relatively small freeze–thaw-induced damage under the same number of freeze–thaw cycles. This finding is consistent with the results of the previous unconfined compressive strength and dynamic compressive resilient modulus tests.

#### 3.3.2. EA-Modified CSG Pore Structure and Microstructure

**Figure 9:**
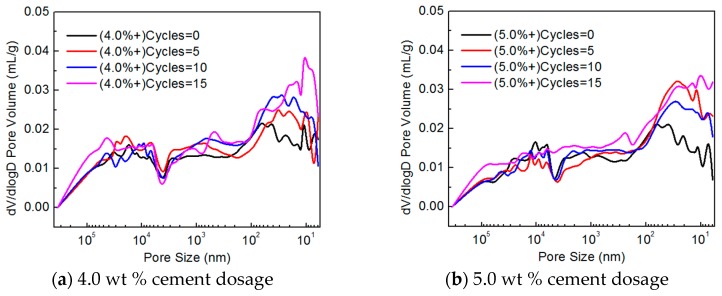
Pore size distribution of cement emulsified asphalt stabilized gravel.

Figure 9 shows the pore size distributions of the EA-modified CSG specimens with various cement contents. As seen, the number of pores in the EA-modified CSG increased as the number of freeze–thaw cycles increased. In contrast to the unmodified CSG, the number of pores in the EA-modified CSG increased to a relatively small extent as the number of freeze–thaw cycles increased, with most of the pores being below 50 nm in size.

A comparison of Figure 7 and Figure 9 shows that the increase in the number of pores with a size in the range of 1 μm–100 nm for the EA-modified CSG, which corresponds to the size range for which the number of pores in the unmodified CSG changed significantly with the number of freeze–thaw cycles, was insignificant as the number of freeze–thaw cycles increased. Based on the mechanism by which the strength of a stable material mixed with EA is formed, the abovementioned phenomenon occurred mainly because EA, compared to water, can more easily react with the aggregates and encapsulate them after binding, and the resulting accumulated pressure will cause the water to be drained away from the surface of the aggregates.

**Figure 10:**
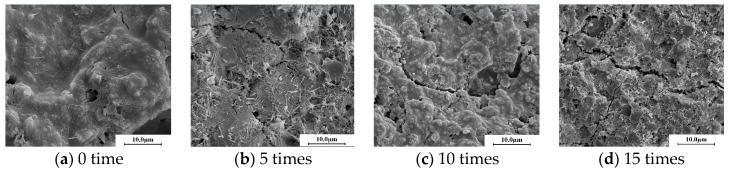
Microstructure of cement emulsified asphalt stabilized gravel.

Figure 10 shows the microstructural morphology of the EA-modified CSG specimens. Acting as a cementing agent, EA encapsulates the aggregates, thereby reducing the permeability and improving the frost resistance of the structure.

The presence of EA could reduce the permeability at the aggregate–cement paste interface and may mitigate the freezing-induced expansion stress generated in the structure during freeze–thaw cycling, which may reduce the rate of formation and number of microcracks.

By comparing the results of freeze-thaw resistance and microstructure, it can be found that with the pore volume increasing, both the strength loss ratio and compressive resilient modulus loss ratio increased. It indicates that denser microstructure can enhance the freeze-thaw resistance of cement-stabilized gravel materials, with or without emulsified asphalt. This also can be verified by observing the microstructure of cement-stabilized gravel materials in Figure 8 and Figure 10. It can be seen from Figure 8 and Figure 10 that more micro cracks appeared as the number of freeze-thaw cycles increased. This led to the reduction of freeze-thaw resistance.

## 4. Conclusions

The objective of this study was to investigate the effect of EA on the freeze-thaw resistance of CSG by conducting mechanical tests and microstructure analyzation. The following is a summary of conclusions that can be drawn based on the aforementioned results and discussion:

The EA-modified CSG with a cement content of 5.0% exhibited relatively good mechanical performance and frost resistance under freeze–thaw cycling. The stiffness and flexibility of the CSG could be adjusted by varying the cement–EA mixing ratio. The addition of EA slightly reduced the initial strength of the CSG but lowered its strength loss ratio and improved its frost resistance.

By encapsulating the aggregates and via its cementing action, EA could reduce the permeability at the aggregate–cement paste interface and, to a certain extent, mitigate the freezing-induced expansion stress resulting from the phase change of the water. Additionally, EA could improve the impervious performance of the base and reduce the rate of formation and the number of microcracks, which is essentially why EA reduced the compressive strength and dynamic compressive resilient modulus loss ratios under freeze-thaw cycling.

## Figures and Tables

**Table 1:** Properties of the ordinary Portland cement P.O42.5.

Items		Test Values	Technical Requirements
Stability (boiling method)		Qualified	—
Initial time (min)		165	≥90
Final setting time (min)		203	≤600
3 days curing	Compressive strength (MPa)	26.4	≥17.0
	Flexural strength (MPa)	5.87	≥3.5
28 days curing	Compressive strength (MPa)	49.6	≥42.5
	Flexural strength (MPa)	8.07	≥6.5

**Table 2:** Properties of the aggregates.

Properties	Test Values	Technical Requirements
Crushing value	13.73	<30%
Abrasion value	8.75	/
Liquid limit (%)	24	<28%
Plasticity index	8	6–9
Clay content (%)	1.76	/
Flat and elongated particles content (%)	5.85	<20%

**Table 3:** Basic properties of emulsified asphalt.

Property	Index Requirement	Test Result
Remaining amount on 1.18 mm sieve (by mass)/%	<0.1	0
Residue content (by mass)/%	58–63	61
Penetration (25 °C, 100 g)/(0.1 mm)	60.0–120.0	76.6
Solubility in trichloroethylene (by mass)/%	>97	99.7
Softening point/°C	–	45.8
Ductility (15 °C)/cm	≥50.0	69.7
Emulsion breaking speed	Slow crack	Slow crack

**Table 4:** Dosage of cement and emulsified asphalt in different samples.

Sample Number	Dosage of Cement (wt %)	Dosage of Emulsified Asphalt (wt %)
A0	4.0	0
A1	4.0	2.5
B0	5.0	0
B1	5.0	2.5

**Table 5:** The variation coefficient of unconfined compressive strength (%).

Freeze-Thaw Cycles (Times)	4.0 wt % Cement Dosage		5.0 wt % Cement Dosage	
	A0	A1	B0	B1
0	4.298	3.871	4.012	3.999
5	4.715	4.210	4.528	3.641
10	4.485	4.399	4.234	4.200
15	5.123	5.620	5.017	4.485

**Table 6:** The variation coefficient of dynamic compressive resilient modulus (%).

Freeze-Thaw Cycles (Times)	4.0 wt % Cement Dosage		5.0 wt % Cement Dosage	
	A0	A1	B0	B1
0	7.910	6.800	6.002	5.377
5	6.798	7.038	7.643	6.090
10	8.100	7.927	7.225	6.358
15	9.203	8.211	7.866	6.732
